# ‘Why, Can I Not Have Control of My Own Insulin?’: Qualitative Exploration Amongst Older Adults With Diabetes With Lived Experience of Surgical Hospital Admission

**DOI:** 10.1111/hex.70509

**Published:** 2025-12-08

**Authors:** Christina Lange Ferreira, Hellena Habte‐Asres, Angus Forbes, Kirsty Winkley

**Affiliations:** ^1^ Care in Long Term Conditions, Faculty of Nursing, Midwifery & Palliative Care King's College London London UK; ^2^ Diabetes and Endocrinology, Hereford County Hospital Wye Valley NHS Trust Hereford UK

**Keywords:** diabetes, frailty, inpatient safety, insulin safety, older adult, perioperative

## Abstract

**Aim:**

To explore the experiences and perspectives of older adults living with diabetes, in relation to insulin management during a surgical hospital admission and identify factors that appear to be associated with insulin safety in these older people. To identify key messages that address people with diabetes priorities, to be used in staff education.

**Methods:**

Qualitative study, conducted in a single, rural NHS hospital. Ten semi‐structured phone interviews were held with older adults with diabetes, with or without frailty, treated with insulin while undergoing surgical hospital admission. Framework analysis was employed in data analysis.

**Results:**

Six people with type 2 diabetes and four people with type 1 diabetes, aged 68 to 91 years old (mean age 77.2 years [SD = 6.2]) participated in the study. Four themes identified specific to hospital insulin management and safety: (1) preparedness for hospital admission; (2) feeling disempowered in hospital; (3) staff knowledge, confidence, attitudes and behaviours; (4) diabetes and insulin management as a balancing act. In addition, participants co‐designed an infographic to help hospital staff understand what matters most to older adults in terms of managing their insulin safely in the hospital.

**Conclusion:**

Lack of joined‐up care, staff knowledge gaps and lack of empowerment ethos hinder hospital insulin safety. This study identified that having a self‐management policy in a hospital does not necessarily mean it is implemented for older adults with diabetes. Using a co‐design approach to engage with older adults helped to identify components critical to hospital insulin safety interventions.

**Patient or Public Contribution:**

Patients and members of the public are involved in study design and interpretation of findings. Before the study, a diabetes peer‐support group reviewed and contributed to the interview topic guide and study recruitment documentation. Study participants co‐designed the infographic developed.

## 1 Introduction

Many people with diabetes use insulin therapy, a subcutaneous injectable treatment that regulates blood glucose levels. There are many different types of insulin with different times of onset, peak and duration, and a range of administration devices and monitoring technology. Doses are tailored to meet individual requirements and usually self‐managed by the person with diabetes or supported by family members or carers [1, 2].

Insulin is considered a high‐risk medication due to the possible harm that can occur in the case of an error [1, 2]. Errors can lead to hypoglycaemia or hyperglycaemia. Untreated hypoglycaemia can lead to coma, seizures and death. Hyperglycaemia has associated risks of infection, delayed healing, osmotic symptoms and hyperglycaemic emergencies: diabetic ketoacidosis (DKA) and hyperosmolar hyperglycaemic state (HHS). Insulin is a time‐critical medication, with a narrow therapeutic window [1, 2].

Most people with diabetes who are admitted to the hospital are not admitted because of their diabetes. This means they are mainly cared for by non‐diabetes specialties, which may lack knowledge and confidence in diabetes management [1]. Ensuring the safe management of subcutaneous and intravenous insulin in hospitals has long been recognised as a complex process, given the multiple components of treatment and the unpredictable environment of healthcare settings. Insulin errors occur frequently in hospitals, at all stages of the medication use process, and can negatively affect inpatient outcomes and experience [1, 3, 4, 5, 6, 7]. Inpatient experience surveys in England (mean age 60.1 [SD, 17.1]) [8] and Australia (2019 data mean age 65.9 SD, 15.1] and 2021 data mean age 68.5 [SD, 14.7]) [9] have identified areas for improvement regarding meal choices and timings [8, 9], inpatient hyperglycaemia and hypoglycaemia [8] staff knowledge [9] and increased facilitation of self‐management of insulin [8, 9]. Being able to self‐manage insulin in hospital was independently associated with greater inpatient satisfaction (*p* < 0.006) [8]. There is a literature gap in understanding the experiences of older adults managing insulin in the hospital.

The perioperative pathway for people with diabetes is particularly complex, involving multiple care teams who work within and outside of the hospital setting [1, 10, 11]. Challenges for staff managing diabetes care in an acute environment include having an inadequate knowledge of diabetes management and insulin administration devices, meeting patient self‐management needs, and facing operational pressures and the time required to provide holistic care [2, 4, 11, 12]. Implementing joined‐up multidisciplinary approaches to optimise the perioperative pathway for people with diabetes in hospital has been shown to significantly improve patient outcomes [10, 11], including reducing hospital length of stay, postoperative complications, diabetes‐related harms and 30‐day readmission [11, 13]. Multi‐component interventions which have included whole pathway approach [11, 14] specific guidelines [15], staff education [11, 14, 15], enhanced communication [11, 14, 15], early identification and optimisation [11, 14, 15], in‐reach diabetes specialist team input [11, 14, 15] and patient empowerment [11] have led to reduction in reported insulin errors [1, 15] and reductions in hypoglycaemia [11, 14, 15] and hyperglycaemic events [11].

However, variations in care and implementation gaps are known challenges [1, 7, 11]. Internationally, diabetes inpatient care audit data has shown the majority of patients in hospital with diabetes are older adults [6, 16, 17]. Hospitalised older adults are likely to be more vulnerable to insulin errors, given the higher prevalence of them having multiple long‐term conditions, polypharmacy and nutritional, cognitive and functional impairments [18, 19]. Older adults with diabetes are also more at risk of having frailty, which is associated with increased risks of postoperative complications and length of hospital stay [18]. Although there are minimal international data that can confirm error occurrence by age range, the National Diabetes Inpatient Audit (NADIA) confirmed that 65% of insulin errors in English hospitals occurred while treating people aged over 65 years [16].

Older adults are also a heterogenous group with a diverse range of physical and mental capabilities, with differing needs in terms of their ability to self‐manage insulin, and who therefore require tailored, individualised diabetes care [18, 20].

There is a clear need to develop and tailor theory‐driven interventions to address hospital insulin safety for older adults. Newer approaches to patient safety suggest that by increasing our understanding of everyday work in practice and considering how safe adaptations can lead to successful outcomes, proactive approaches to safety can be built [21, 22]. Healthcare comprises dynamic complex, adaptive systems with multiple interacting components, characterised by nonlinearity, unpredictability and emergent behaviours [22, 23]. In these contexts, solely relying on more traditional, linear reactive responses to harm (known as ‘Safety‐I’ principles) has been shown to have limited impact on patient safety [22]. More recent research employing a ‘Safety‐II’ approach suggests that better understanding everyday work and the differences between work‐as‐imagined and work‐as‐done can help build adaptive capacity to support safe practice despite the obstacles and difficulties in a dynamic system [21, 22, 24]. Resilient healthcare approaches also recognise that in complex systems, there is a need to consider systems‐based approaches to safety which build capacity to handle complexity and variable conditions [22].

Despite older adults with diabetes being most affected by and at risk of insulin errors in hospital, there has been limited in‐depth exploration of their experiences of insulin management during surgical hospital admissions and of their involvement in the development of insulin safety interventions.

The study reported in this paper is part of a wider co‐design study: Developing an intervention for safe hospital insulin use amongst older adults with diabetes, with or without frailty, undergoing a surgical hospital admission (SHINE) [25]. Following the MRC Framework for complex interventions [26], the project employs participatory methods, through a co‐design approach [27], and uses design thinking principles [28] to encourage collaborative working between staff and people with diabetes [27, 29].

The SHINE study has two main phases. An exploratory ‘inspiration’ phase amongst older adults with diabetes and multiprofessional staff to explore experiences and perspectives. Then, an ideation phase, which brings together people with diabetes and staff in a co‐design process to work collaboratively on developing an intervention for safer hospital insulin use. This paper reports on the qualitative study held with older adults with diabetes, which formed part of the ‘Inspiration’ phase for SHINE [25] and which provided a foundation for subsequent intervention development in ‘Ideation’ phase.

Co‐design has been used successfully to develop a range of innovations to improve healthcare [27, 29]. Creative input from professional graphic designers to support the communication of key information has enhanced the co‐design process and outputs developed [30]. There is increased interest in the incorporation of the arts in its many forms, to improve health‐related communication [31] and in the education of healthcare professionals, as a way of promoting empathy and reflective practice [32].

This qualitative study therefore had the following aims:

- To explore experiences and perspectives of older adults with diabetes, with or without frailty, in relation to insulin use during a surgical admission.
- To identify key factors in practice that appear to be associated with hospital insulin safety and insulin errors in this population and setting.
- To identify key messages participants would like to relay to hospital staff on the topic ‘what matters to me for safe insulin management in hospital’ and co‐design an illustrative infographic capturing these messages.

## 2 Methods

### 2.1 Study Design

A qualitative study employing telephone semi‐structured interviews was conducted. Subsequent feedback activities with participants contributed to the co‐design of an illustration infographic with key messages identified by participants.

Reporting adhered to Standards for Reporting Qualitative Research (SRQR) [33]. National Health Service (NHS) Health Research Authority ethical approval from East Midlands‐Derby Research Ethics Committee (24/EM/0022) was gained before study commencement.

### 2.2 Setting

This single NHS hospital site study was carried out at a local general hospital in a rural county serving a population of 235,000 people in both England and Wales. The hospital had a multidisciplinary (medical, nursing and podiatry) inpatient diabetes specialist team working Monday to Friday. There was a perioperative pathway in place based on national guidance [10]. There had also been a recent attempt at the time of data collection to introduce a ‘perioperative diabetes passport’ [11], which was a resource shared by hospital staff and patients with key information about an individual's diabetes management. The hospital also had a stated policy to support patient self‐management of insulin.

### 2.3 Participants and Sampling

People with diabetes were purposively recruited based on the following eligibility criteria: (1) diagnosis of diabetes before hospital admission at the research site who used insulin before and during their hospital stay, (2) aged 65 years or older (3) with a history of hospital admission for general or orthopaedic procedures at one of 4 designated wards, requiring a minimum of one night hospital stay postoperatively within the last 9 months (4) who were able to give informed consent (5) who had sufficient English to undertake the interview. Recent admission in the last 9 months was selected as the inclusion criterion to provide participants with a sufficient level of convalescence post‐admission, but recent enough to allow accurate recall and ensure the relevance of their experience. The wards were selected as these were where staff experiences were also being explored as part of the SHINE study [34].

From a pool of eligible patients, purposive sampling was facilitated through the assistance of the diabetes specialist team (the Diabetes consultants and Diabetes specialist nurses). This enabled the identification of people with different types of diabetes and insulin treatment modalities, duration of diabetes, varying levels of frailty measured using the Clinical Frailty Scale (CFS) [35], and different durations of hospital admission.

The concept of information power [36] was used to guide sample size during the interview phase. Information power considers study aims, sample specificity, use of established theory, the quality of dialogue and analysis strategy as suitable criteria for sample size assessment. The greater the information a sample holds of relevance to the study, the lower the number of participants required. The research team continuously assessed information power throughout the data collection process and preliminary analysis. In view of the focused study aims, rich data provided, high specificity of the study population to the study aims and use of a priori knowledge and framework analysis, information power to meet the study aims was considered sufficient with the final sample of 10 participants.

### 2.4 Recruitment

The study was advertised using posters on various wards, via clinical teams and through partnership local organisations (e.g. Healthwatch) who disseminated study information through social media. Potentially eligible participants were identified by the diabetes specialist team in routine practice or by retrospective review of admission data. Participants could also self‐refer if they were interested in taking part. All potentially eligible/interested participants were screened by the lead researcher (CLF) either face to face or remotely to ensure they met the inclusion criteria. Participants were provided with patient information and given written consent if they wanted to take part. It was emphasised that they were under no obligation to participate and that their clinical care would not be affected should they decline the invitation or later withdraw from the study. Participants were offered a £15 Amazon voucher to thank them for their time. Participants were offered a choice of face‐to‐face, telephone or Microsoft Teams interviews.

### 2.5 Data Collection

An initial topic guide for the interviews was developed by the team. This was informed by a previous scoping review undertaken, which had included development of the RESILIENT Framework to explore and classify insulin errors in hospital [3], which drew from a human factors framework Safety Engineering for Patient Safety (SEIPS) [37] and from the World Health Organization strategic framework for the global patient safety challenge: Medication without harm [38]. It was then reviewed and refined by a Patient Participation Involvement (PPI) group of people with diabetes for clarity and content. A pilot interview was conducted with one person, but no changes were made to the interview topic guide as a result. The interview asked participants about the advice they had received in preparation for elective surgery (if relevant) and explored their experiences, perspectives and challenges of insulin use, self‐management and insulin errors during admission. It also asked for their ideas on improving hospital insulin safety for older people, with or without frailty. The interview topic guide can be found in Appendix 1.

All participants chose to be interviewed by telephone. The interviews were held between April to October 2024, lasted between 20 and 60 min (median 34.5, interquartile range 14.25) and were conducted by the lead researcher (CLF). The lead researcher (CLF) is a diabetes specialist nurse, trained in qualitative interview techniques, who has worked clinically at the research site. To minimise any potential bias, a team approach was taken across data analysis and interpretation. Team members, HH‐A, AF and KW are all clinical‐academic diabetes specialist nurses possessing a range of clinical and research experience. Although all were in clinical practice, none worked at the study site. Consideration of trustworthiness criteria in qualitative research; credibility, dependability, confirmability and transferability [39] guided the study and the processes by which it was ensured are shown in Table 1.

**Table 1:** Steps undertaken in framework analysis process.

Step in framework analysis process	Description of activities	Consideration of Lincoln and Guba [39] trustworthiness criteria in qualitative research
1. Familiarisation	CLF listened to the full audio of all interviews and checked the transcripts for accuracy. KW and HH‐A listened to portions of the audio from all 10 interviews to achieve familiarisation with the data. CLF read and re‐read the full transcripts to achieve familiarisation with the data. Portions of transcripts were read and re‐read by the research team (HH‐A, AF, KW) at regular meetings during the study period.	Credibility: in depth interviews generated rich data which the study team familiarised themselves with. There was analytical discussion and debriefing with the research team.
2. Identifying a thematic framework	A framework was developed using an inductive‐deductive process. CLF undertook line‐by‐line coding of 4 interview transcripts selected from interviews with rich, deep data and representing different participant characteristics such as sex, type of diabetes and type of admission (elective or emergency). This generated 40 initial codes. Combining previous knowledge and theory from Complex Systems thinking, Safety 2 and Resilient Healthcare principles [21, 22, 23] and a scoping review previously undertaken by the research team to identify insulin errors and associated interacting components [3] an initial framework with 6 categories (Access to insulin; Staff knowledge attitudes and behaviours, Empowerment vs disempowerment, Emotions, Balancing act) each with subcategories was developed. This was reviewed and discussed by the research team (HH‐A, AF, KW). Appendix 2 details sample preliminary codes, thematic framework developed and final themes and subthemes.	Dependability: Iterative process of framework development underpinned by previous guidance in using and applying framework analysis [40, 41, 42] Regular discussion and reflection amongst the study team.
3. Coding and indexing	A code book was developed, and the thematic framework was applied to the data by CLF using NVivo 14 software. HH‐A, AF and KW regularly reviewed transcript portions during the analysis process. This provided additional perspective, opportunity to discuss different interpretations of the data and confirmed usability of the coding framework. HH‐T coded portions of four transcripts and coding agreement was noted. The ‘other’ code [41] was used for each category to ensure data that may not fit the framework was not missed.	Use of a codebook provides a transparent process with a clear audit trail, which enhances dependability of the study. Reflexive journals documented CLF's thoughts, ideas and personal interests. These were discussed amongst the research team to identify any personal biases.
4. Charting	After completion of coding, CLF entered the data into framework matrices for each theme.	Dependability This allowed for patterns, connections and ideas to be considered across the data.
5. Mapping and interpretation	Themes were mapped and interpreted and became the results of the study.	Confirmability: Study findings are supported with anonymized extracts of participants contributions.

### 2.6 Data Analysis

#### 2.6.1 Interview Data Analysis

Audio files were transcribed by professional transcription services. Transcripts were not returned to participants for review before analysis. NVivo 14 software was used to manage the data.

Framework analysis was used to analyse the interview data [40, 41, 42]. This approach allowed for the incorporation of a priori knowledge from a previous scoping review undertaken [3] and prior theoretical concepts from complex systems thinking [23], Safety‐II [21, 22] and Resilient Healthcare [22]. The application of the five integrated phases of the framework analysis process (familiarisation, identifying a thematic framework, coding and indexing, mapping, interpretation) are fully described in Table 1. A sample of preliminary codes, the thematic framework applied to the data, and how this relates to the final themes and subthemes are represented in a diagram in Appendix 2.

#### 2.6.2 Codesign of Infographic: ‘What Matters to Me’

The infographic's content was co‐designed with participants through the interviews and follow‐up validation and refinement. During the interviews, participants were asked to identify key messages that they would like included in an infographic for staff, which aimed to relay ‘what matters to me in hospital insulin management’.

The research team formulated participants’ key messages into eight statements. CLF met with a graphic designer to develop a draft infographic. This received initial validation and refinement from two participants who had been interviewed and were attending a planned face‐to‐face workshop related to the ‘ideation phase’ of the SHINE study. The other 8 participants declined attendance. The workshop was held in an accessible venue outside the hospital site. A second draft of the infographic was sent to all ten interviewees for feedback by post. Six participants responded with final comments. These comments informed changes to wording of messages, and the order and content of the illustrations (examples in Appendix 3). CLF, HH‐A, AF and KW met to discuss this feedback and CLF liaised with the graphic designer to produce the final version of the infographic. This was then validated at another workshop meeting attended by two of the people with diabetes interviewed.

The intention is for this to be disseminated to health professionals at a future stage of the study and used as a tool in staff education at the research site. It will also be made available to other organisations.

### 2.7 Reflexivity

The interviewer CLF (MSc, female) has prior clinical experience as a diabetes specialist nurse and has provided care to people with diabetes in an inpatient and outpatient setting. The other members of the research team (HH‐A, AF and KW) are all clinical academics with clinical backgrounds in diabetes and experience in mixed methods research, including qualitative studies.

## 3 Results

### 3.1 Sample Characteristics

Ten older adults with diabetes were recruited and interviewed. The mean age of participants was 77.2 (SD = 6.2) (range 68 to 91 years). All identified their ethnicity as White and were native English speakers. Seven were identified as female and the remainder as male. Diabetes duration ranged from just over 10 to 61 years. Four had type 1 and six had type 2 diabetes and all used insulin pens and self‐managed their insulin treatment before admission. Sample level of frailty ranged from ‘managing well’ to ‘living with moderate frailty’ on the clinical frailty scale v2.0 [35]. Participant characteristics are summarised in Table 2.

**Table 2:** Sample characteristics.

Participant number	Age	Sex	Ethnicity	Occupation and usual place of residence, civil status	Highest level of education	Type and duration of diabetes	Type of insulin and devices, Method of glucose monitoring and level of independence with insulin treatment	Reported hypoglycaemia awareness (Can you feel your hypos coming, at what level and what symptoms)	Level of frailty Clinical frailty scale [35]	Elective or emergency	Surgical specialty and admission duration	Number of hospital admissions in last 12 months
1	77	Female	White	Retired, Own home, married	Left school age 15	Type 1 diabetes, duration 23 years	Basal bolus, prefilled insulin pens Libre 2 and glucometer Independent	Reported Impaired awareness of hypos	Living with mild frailty	Emergency	Geri‐Orthopaedics 65 days (including community hospital stay)	6
2	73	Male	White	Retired, Own home, married	Left school age 15	Type 1 diabetes, duration 56 years	Basal bolus, prefilled insulin pens Libre 2 and glucometer Independent	Reported Impaired awareness of hypos	Living with mild frailty	Elective	Orthopaedics 4 days	1
3	68	Male	White	Retired, own home, divorced	O levels	Type 1 diabetes, uration 40 years	Basal bolus, insulin cartridges Libre 2 and glucometer Independent	Reported good level of awareness	Managing well	Elective	Orthopaedics 3 days	1
4	72	Female	White	Retired, own home, divorced	O levels	Type 1 diabetes, duration 61 years	Basal bolus, insulin cartridges Libre 2 and glucometer Independent	Reported some awareness	Managing well	Elective	Orthopaedics 10 days	1
5	79	Female	White	Retired, own home, divorced (lives alone)	Left school age 15	Type 2 diabetes, duration 25 years	Basal bolus, prefilled insulin pens Libre 2 and glucometer Independent	Reports good awareness	Living with very mild frailty	Elective	Orthopaedics 3 days	4
6	85	Male	White	Retired, Own home, married	University	Type 2 diabetes, duration 20 years	Biphasic insulin, three times daily, prefilled insulin pens Libre 2 Independent	Reports good awareness	Living with very mild frailty	Emergency	Geri‐Orthopaedics 3 days	1
7	77	Female	White	Retired, own home, widow (lives alone)	Professional Registration	Type 2 diabetes, duration 24 years	Basal insulin, prefilled insulin pens Glucometer Independent	Reports never had hypos	Managing well	Elective	General surgery 2 days	1
8	74	Female	White	Retired, own home, single (lives alone)	Tech college	Type 2 diabetes, duration over 10 years	Basal insulin, insulin cartridges Glucometer Independent	Reports good awareness	Managing well	Elective	General surgery 3 days	2
9	91	Female	White	Retired, own home, widow (lives alone)	Left school age 16	Type 2 diabetes, duration over 30 years	Intermediate insulin, prefilled insulin pens Glucometer Independent	Reports good awareness	Living with moderate frailty	Elective	General surgery 2 days	2
10	76	Female	White	Retired, own home, widow (lives alone)	University	Type 2 diabetes, duration over 10 years	Basal insulin, prefilled insulin pens Glucometer Independent	Reports impaired awareness of hypos	Living with very mild frailty	Elective	General surgery 6 days	1

### 3.2 Qualitative Findings

**Figure 1:**
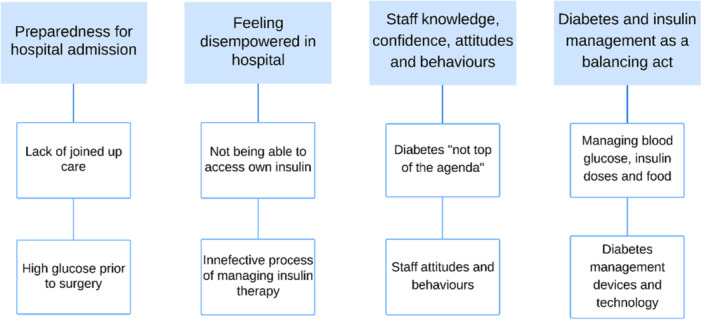
Themes and subthemes emerging from the data. Figure 1 **Alt text:** Graphical representation of the four main themes and associated subthemes which emerged from the data: Preparedness for hospital admission; Feeling disempowered in hospital; Staff knowledge, confidence, attitudes and behaviours; Diabetes and insulin management as a balancing act.

Findings are presented under four main themes (and subthemes) identified from the data and are presented in Figure 1. These are: (1) Preparedness for hospital admission; (2) Feeling disempowered in hospital; (3) Staff knowledge, confidence, attitudes and behaviours; (4) Diabetes and insulin management as a balancing act.

Representative participant quotes can be found below in the narrative synthesis with additional quotes in supporting material Appendix 4.

**Theme 1: Preparedness for hospital admission**

Participants reflected on their assumptions, expectations and preparedness for hospital admission and identified areas for improvement. Two sub‐themes were identified:

**Subtheme: Lack of Joined‐up Care**

Two participants experienced emergency admissions for trauma‐orthopaedic reasons. With little preparation beforehand, they spoke about a backdrop of organisational pressures, a lack of joined‐up thinking and ‘corridor care’ while awaiting a bed, all of which they felt had a negative effect on the safety of their insulin management.

Whatever happened took a long time to get my insulin… It worried me a lot. It made me anxious, is the word. (P1, 77 years living with Type 1 Diabetes Mellitus (T1DM))

All participants undergoing elective procedures, rather than emergency admissions, had their diabetes and insulin treatment acknowledged in consultations before surgery. However, the quality of information received before surgery appeared to vary. Some participants worried about the accuracy of the dose adjustment advice received pre‐operatively. Others wanted more detail about the use of intravenous insulin infusion in hospital but as one participant highlighted ‘*nobody seemed to know who should be doing it*’ (P4).

Crucially, the possibility of being able to self‐administer insulin in hospital was not discussed with them:

Why was I never told about this [possibility of self‐administration of insulin] from the beginning? Because that would've so made me feel less stressed about it all. (P3, 68 years old living with T1DM)

**Subtheme: High glucose before surgery**

Some participants experienced hyperglycaemia before surgery, contributing to delays in treatment. Staff did not always appear to know whether to administer insulin or not in such circumstances. Some participants would have preferred to have been given the choice to have intravenous insulin infusion during surgery and immediately postoperatively. However, a lack of a firm plan about if, where and when to start an IV insulin infusion, led to delays on the day of surgery and increased stress:

So, the day of surgery, their operating started two hours late because of it. So, that made me feel a bit stressed. (…) I remember saying at one point my blood sugar is now up to 17 [mmol/L]… (P4, 72 years old and living with T1DM)

**Theme 2: Feeling disempowered in hospital**

Several participants talked about a range of interacting issues during their hospital admission, which led to them feeling disempowered in hospital. Two sub‐themes were identified.

**Subtheme: Not being able to access own insulin**

Participants described how the process of admission to the ward involved their medications, including insulin, being taken away from them and managed by the nurses.

And when I got to the ward, they had taken my medication and they had locked it away. (P5, 79 years old, living with Type 2 diabetes (T2DM))

While staff sometimes asked for advice from the person with diabetes on how much insulin they should administer, participants were frustrated that they did not give them full control over the treatment:

People are capable of doing their own insulin. I don't think it matters if you're young or old, you know. We understand our bodies better than what they do, and we understand about the diabetic side. (P10, 76 years old living with T2DM)

Conversely, a few participants were either too unwell to discuss self‐management with staff or were happy to have the nurses in charge of their insulin treatment, one participant described that they ‘*had faith in them*’ (P8) and another that it was ‘*nice to be waited on*’ (P9).

Participants appreciated that there were occasions when it was necessary for staff to manage the insulin:

on the day of the operation (…) [staff] need to manage (…) [insulin] in case you are not completely with it. (P3, 68 years old living with T1DM)

However, regaining control of their insulin therapy was described by a few participants as a ‘*battle’* (P4). Some participants were told initially they were ‘*not allowed’ (*P3) to self‐manage, and others found their insulin was still inaccessible even if they had ‘*signed a [consent] form*’ (P2) to be able to do so, as part of the policy on self‐management of insulin in hospital.

And in the end I said, ‘Why when I've been diabetic for 40 years can I not have control of my own insulin so that I can get my blood sugar down?’ and they said, ‘It's hospital policy you can't do that. (P3, 68 years old living with T1DM)

**Figure 2:**
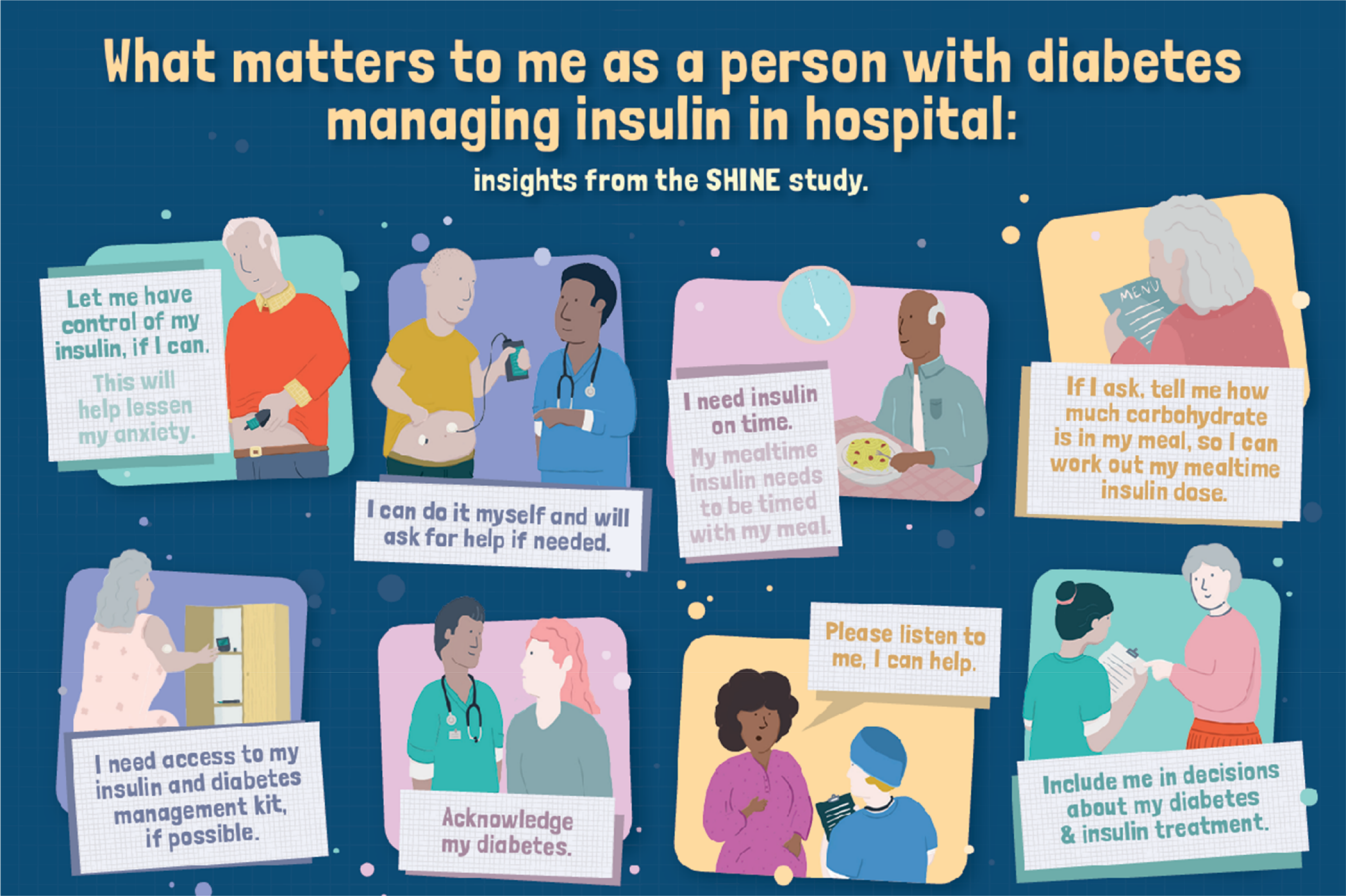
Co‐designed Infographic: ‘What matters to me’. Figure 2 **Alt text:** Eight key messages that participants want to convey to healthcare professionals working in hospital on the topic ‘what matters to me managing insulin in hospital’ are represented in illustrations combined with text: Let me have control of my insulin if I can; I can do it myself and will ask for help if needed; I need insulin on time; If I ask, tell me how much carbohydrate is in my meal, so I can work out my mealtime insulin dose; I need access to my insulin and diabetes management kit, if possible; Acknowledge my diabetes; Please listen to me, I can help; Include me in decisions about my diabetes & insulin treatment.

Participants wanted the infographic developed [Figure 2] to emphasise to staff the importance of consulting them about their preferences for self‐management.

Because when you've been a diabetic for 40 years, everything revolves around your care, so insulin is a big, big, big, big thing in that. And if somebody comes along and takes it away from you, it's like having your arms chopped off, it's horrible. (P3, 68 years old living with T1DM)

Messages included: ‘*let me have control of my insulin*’ and ‘*include me in decisions about my diabetes and insulin treatment*’. It was equally important that they felt they could always ‘*ask for help if needed*’ if their ability or desire to self‐manage fluctuated during admission.

**Subtheme: Ineffective Process of Managing Insulin Therapy**

Recognising ‘*how busy the nursing staff are*’ (P4), many participants described how their injections were ‘*always late*’ (P1). Significant variations in their glucose levels were a source of upset and worry among participants.

They checked my sugar levels at about 6.30 in the morning. Breakfast came (…) 8 o'clock, still not allowed to have my insulin. (…) at about 9.30 and then I was allowed to have it, but it was [then] locked away. By then I was very stressed out. (P5, 79 years old living with T2DM)

In the infographic, older adults were keen to include how having access to their insulin reduced their anxiety (Figure 2).

Several participants experienced hyperglycaemia postoperatively. They explained how it was a time‐consuming process to raise the issue with the nurses, for the doctor to then review, prescribe and authorise additional insulin.

Because I always had to wait for a doctor's permission for the insulin and the insulin had to be given by the nurse, it was all, in my opinion, very wrong. (…) So everything they did all took time; time which I was concerned for an infection starting. (P3, 68 years old living with T1DM)

Participants observed that having hyperglycaemia postoperatively sometimes led staff to ‘*panic*’ (P5). Those participants who eventually were able to self‐manage their insulin therapy in hospital reported that they got their ‘*levels down quite quickly*’ (P3).

**Theme 3: Staff knowledge, confidence, attitudes and behaviours**

Reflecting on general aspects of their hospital admission, surgery and care, several participants felt very pleased:

hospital care was wonderful (…) the staff were all brilliant. (P3 68 years old living with T1DM).

It was aspects of their diabetes care and insulin management that had caused distress during their admission.

**Subtheme: Diabetes** ***‘*** **not top of the agenda’**

A few participants felt that for most professionals looking after them, diabetes ‘*wasn't top of the agenda*’ (P10). While not expecting it to be the focus during a surgical admission, participants were clear that how their diabetes had been managed had had a significant impact on their postoperative recovery. Hence one of their messages to staff in the infographic was ‘*acknowledge my diabetes*’ (Figure 2).

Participants felt that staff looking after them often did not have enough knowledge about diabetes and insulin management. In the infographic, they wanted to stress that staff could use them as a reliable source of information on insulin management; ‘*please listen to me, I can help*’ (Figure 2).

Some voiced concern about nurses’ lack of confidence with insulin administration techniques leading to underdosing and consequently, persistent hyperglycaemia.

So she [nurse] took the needle off, put a new needle on, did it again, and I said, ‘Hang on a minute, you're not charging the needle with the insulin. There's an air gap in here. So what you've effectively done is injected me with air,’ (….) and I showed her. (…) So I said, ‘Do you see now what the problem is? You've been giving me these little doses but there's nothing in it so you're not actually giving me anything unless the needle (…) is charged with insulin. (P3; 68 years old living with T1DM)

Participants did not ‘report’ these types of errors; as one participant put it ‘*because I didn't want to make a fuss*’ (P3).

**Subtheme: Staff attitudes and behaviours**

Participants reported different experiences regarding communication by staff and their attempts to involve people in their own diabetes management. How effective staff were at this affected participants’ experiences either positively or negatively:

they were doing the testing, but they weren't telling me what it was, if it was high, low or…(…) I felt a bit anxious, you know, because I like to keep on top of things if I can… (P10, 76 years old living with T2DM)

Several messages in Figure 2 relay the value placed on staff attitudes and behaviours that foster open communication, partnership and empowerment. Participants recalled with gratitude occasions of individual professionals’ ‘*diligence*’ (P4) or ‘*kindness*’ (P9) in supporting them in their diabetes management or reducing their anxiety.

**Theme 4: Diabetes and insulin management as a balancing act**

Participants identified the constant challenge and balancing act of managing diabetes and insulin therapy; as one participant put it: ‘*It's just not a clear cut and dried…do A and B will result*’ (P4).

Two subthemes addressing the intersectionality of glucose and insulin management, food, diabetes management devices and technology were identified.

**Subtheme: Managing blood glucose, insulin doses and food**

Participants explained how surgical‐related pain and stress, inactivity and being restricted to a bed all had different impacts on their glucose profile, making it more difficult to manage than usual. While older adults had access to snacks, most disliked the meals offered by the hospital, which reduced their food intake. They felt that poor staff knowledge of both diabetes and the content of meals made it challenging to eat appropriately. Participants mentioned general comments from catering staff such as ‘*Just don't eat the sweet puddings*’ (P4). In fact, only one participant reported that they had ready access to the carbohydrate content of meals served in hospital.

There was also a frustrating lack of coordination between glucose monitoring, meal delivery and insulin administration with a subsequent impact on patient glucose profiles:

And by lunchtime I was asking for my insulin, ‘Oh, the nurse will be round with it later’. I said, ‘I need to take it with my food!’ and nobody was listening, and I was in tears’. (P5, 79 years old living with T2DM)

In the infographics, participants stressed the importance of ensuring better coordination of mealtimes and insulin administration, and that they should be provided with greater details about the carbohydrates in the hospital food to help them determine their mealtime insulin dose (Figure 2).

**Subtheme: Diabetes management devices and technology**

Participants who used wearable glucose technology found this helpful in supporting their ability to self‐manage insulin in hospital as they had ready access to their glucose data:

I was able to monitor my own [glucose] constantly. (P3 68 years old living with T1DM)

However, some staff were unfamiliar with how this newer wearable glucose technology worked. In the infographic, participants also requested easy access to their diabetes management kit, which included their usual insulin needles, rather than having to use hospital equipment ‘*safety needles*’ (P3), which they were not confident with (Figure 2).

While none of those interviewed were using insulin pump therapy, participants felt it was likely to become more common, and that it was important to raise staff awareness of the range of different insulin devices that patients might use, hence its inclusion in the illustrations.

The final infographic, which was co‐designed with participants, can be seen in Figure 2, summarising the eight key messages which patients wanted directed to hospital staff.

## 4 Discussion

This novel study has identified a number of experiences and challenges faced by 10 older adults with diabetes managing insulin during surgical admission, as described by the older adults themselves. The themes generated from the data provide useful insights to help inform strategies to enhance insulin safety, staff education, patient empowerment and care experience for older adults with diabetes.

Problems with achieving joined‐up working in insulin management during surgical admissions have been confirmed through audit studies and case reviews [1, 43, 44]. In terms of organisation, all elective participants in our study had their diabetes and insulin treatment acknowledged before surgery, with plans for prioritisation on theatre lists, aligning with national recommendations [10]. However, a few participants disclosed that they suffered hyperglycaemia in the lead up to surgery and postoperatively, which is concerning given its association with postoperative complications [10, 45, 46]. Our study also identified patient dissatisfaction with insulin injection timing, which is consistent with other studies in the UK and Australia [12, 47] Equally, there were further problems with emergency admissions, with some patients not bringing insulin into hospital, long waits for a hospital bed and delays in administering time‐critical medication. Organisational pressures and operational capacity in healthcare settings have been previously identified as major contributing factors to insulin errors [3, 12].

Participants wanted to know before admission about the opportunity to self‐manage in hospital, and indeed, were frustrated by the failure of the hospital to empower them to do so once admitted. Not being in control of their insulin or having access to key information about their meals led to upset and anxiety. Participants were aware that their glucose levels were not optimal but felt powerless to manage the situation. The benefits of hospital self‐administration of insulin are well established for both patients and staff across the international literature [12, 47, 48, 49]. Being able to self‐manage insulin in hospital when appropriate and safe is also recommended by international diabetes associations [4, 49]. However, studies in Australia and the UK have acknowledged the wide variation across hospitals in both the availability and use of insulin self‐management and self‐administration policies [2, 5, 6]. Importantly, our study also found that some older adults wanted to leave nurses in charge of their insulin treatment. Equally, inpatient experience surveys in England and Australia have found that not all patients want to take control of their diabetes management while in hospital [9, 13]. Different cultural nuances and preferences can also influence patient assumptions, expectations and behaviours with regard to advocacy and empowerment [50]. Therefore, recognising the heterogeneity of older adult population [20], better communication with older adults is needed, along with flexible self‐management policies to ensure individualised care that considers patient preference and need throughout hospitalisation. A Danish study on a general surgical ward found that older patients (aged over 80) were often not asked about or offered the possibility of self‐administration [51].

Several of the key messages in the infographic for hospital staff reflect the desire of older adults to increase patient empowerment. It is likely that older adults face even greater cultural challenges than the general patient population to making their voice heard in healthcare. Previous research has identified significant service challenges to patient empowerment in older adults with advanced disease in other inpatient settings [52]. Older people, or those with frailty, may also have lower health literacy, impaired hearing and communication skills, greater co‐morbidity and cognitive issues, and may be viewed in a more paternalistic way by staff. This, coupled with an organisational approach that is not patient‐centred or culturally responsive, can hinder older adults’ agency, self‐efficacy, self‐management and choice [50, 52].

Implementing and embedding self‐administration and management of insulin in hospitals is considered a complex intervention. Challenges include changing the culture and staff beliefs regarding patient self‐administration in hospital [48, 53, 54]. Additionally, diabetes specialist teams often lack time and capacity to take on the implementation of complex interventions on top of their usual heavy workload [53]. Dedicated project management time and organisational engagement are known factors for successful implementation [55].

Similar findings relating to staff diabetes knowledge gaps have been reported previously [1, 12, 56, 57]. Improvements to multiprofessional pre‐registration diabetes training and professional development opportunities are needed in a rapidly changing diabetes management landscape [49, 56]. Multicomponent interventions in hospitals in the USA have demonstrated that addressing insulin timing administration by nursing staff through education, process and cultural changes can lead to a reduction in insulin errors [58, 59]. Training and support needs to foster a ‘no blame’ culture, increase collaboration, patient centeredness and familiarity with clinical processes. Such an approach will help learners to apply knowledge to varied and uncertain contexts and to make wiser decisions [56, 57]. Additionally, remote or rural hospitals such as the one recruited in this study, face greater challenges than urban teaching hospitals in terms of recruitment, training and retaining their workforce [60]. If these challenges are not adequately addressed, the quality of diabetes care and services available to their rural populations is likely to be adversely affected.

Of concern is that our participants identified a number of insulin management errors while hospitalised, although they did not formally report any incidents. Capturing the experience of people with diabetes is key in the context of system‐based approaches to increasing resilience in insulin safety. While a recent patient‐reported experience measure for adult diabetes inpatient care has been developed and validated [61], this does not focus on insulin management specifically. Given the complexity of this high‐risk treatment, capturing a more granular picture of insulin management through reported patient experiences could help identify aspects to enhance insulin safety, which may go unrecognised in busy clinical environments.

### 4.1 Strengths and Limitations

This study provides a novel contribution to the literature by reporting the experiences and challenges faced by 10 older adults with diabetes, with or without frailty, when managing insulin during a surgical hospital admission. The study findings identify areas that can be targeted to improve patient care in surgical settings and where future research is needed.

However, this is a single‐site study and thus our findings may not be transferable to other healthcare settings or other countries. While it was a consensus decision based on Malterud et al. [36] that sufficient information power to meet the study aims was met with our sample of 10 participants; however, this is open to interpretation.

The study was successful in recruiting people who are less represented in research, including those who are older, from rural areas, digitally excluded, and living with multiple health conditions. However, there was a notable lack of ethnic diversity in the sample, and further research is required to identify possible additional challenges for minoritised populations.

It is possible that participants who agreed to take part in this study may have had different or more extreme ideas or experiences about insulin safety compared to those older adults who chose not to take part. In addition, all of our participants were self‐managing their insulin on recruitment, and thus were likely to be relatively independent in facilitating their own care. Participants had a duration of diabetes of at least 10 years and were well established on insulin treatment before admission. People with a shorter duration of diabetes and insulin treatment may hold different perspectives. Future research is needed that focuses on the experiences of adults less able to self‐manage their own insulin therapy, and which considers how best to engage this population living with frailty, and their families and carers in research.

Finally, the lead researcher (CLF) worked at the hospital site during the study and was known to one interviewee through their clinical work. While an ‘insider’ position had advantages, such as familiarity with the setting context and the teams supporting recruitment, the potential for bias was appreciated. Therefore, regular reflexivity was practiced by the lead researcher and a team approach was taken to all data analysis and interpretation.

## 5 Conclusion

Participants in this study experienced disjointed inpatient care in relation to their diabetes management, a lack of patient empowerment and variation in staff knowledge and behaviour. This study highlights that having diabetes care guidelines in place does not necessarily ensure successful implementation into practice. Understanding the experiences of older adults, with or without frailty, can help identify key components for future interventions to enhance hospital insulin safety in this population.

## Author Contributions

**Christina Lange Ferreira:** conceptualisation, data curation, formal analysis, investigation, methodology, project administration, visualisation, writing original draft. **Hellena Habte‐Asres:** conceptualisation, formal analysis, methodology, supervision, validation, writing – review and editing. **Angus Forbes:** conceptualisation, formal analysis, methodology, supervision, validation, writing – review and editing. **Kirsty Winkley:** conceptualisation, formal analysis, methodology, supervision, validation, writing – review and editing.

## Ethics Statement

National Health Service (NHS) Health Research Authority ethical approval from East Midlands‐Derby Research Ethics Committee (24/EM/0022) was gained before study commencement.

## Conflicts of Interest

C.L.F. reports financial support was provided by Foundation of European Nurses in Diabetes. C.L.F. reports having received grants towards research costs from Novo Nordisk UK Research Foundation (NNUKRF), Herefordshire and Mid‐Powis Diabetic Care Fund and Florence Nightingale Faculty of Nursing, Midwifery and Palliative Care Staff Development Fund. HH‐A reports having received speaker honoraria from AstraZeneca and Bayer. The other authors declare no conflicts of interest.

## Supporting information


**Appendix 1:** Interview topic guide. **Appendix 1 Alt text:** Text box showing interview topic guide; probing themes and examples of probing questions used in the interviews with research participants. **Appendix 2:** Preliminary codes, initial thematic framework and final themes and subthemes. **Appendix 2 Alt text:** Graphical representation of the analysis and coding process: from examples of preliminary codes to development of an initial thematic framework with 6 categories, which then developed into the four final themes and subthemes and the infographic, which are the results of this paper. **Appendix 3:** How participant contributions informed the co‐design process of the infographic. **Appendix 4:** Additional representative quotes.

## Data Availability

The data that support the findings of this study are available within the article and the supporting material of this article.
