# Supplementary material for: ‘Why, Can I Not Have Control of My Own Insulin?’: Qualitative Exploration Amongst Older Adults With Diabetes With Lived Experience of Surgical Hospital Admission
Source: Health Expect. 2025 Dec 8;28(6):e70509. doi: 10.1111/hex.70509 (PMC12683602; doi:10.1111/hex.70509)
Supplement: Supplementary file 1 — Appendix 1: Interview topic guide. Appendix 1 Alt text: Text box showing interview topic guide; probing themes and examples of probing questions used in the interviews with research participants. Appendix 2: Preliminary codes, initial thematic framework and final themes and subthemes. Appendix 2 Alt text: Graphical representation of the analysis and coding process: from examples of preliminary codes to development of an initial thematic framework with 6 categories, which then developed into the four final themes and subthemes and the infographic, which are the results of this paper. Appendix 3: How participant contributions informed the co‐design process of the infographic. Appendix 4: Additional representative quotes. [file HEX-28-e70509-s001.docx]

"Why, can I not have control of my own insulin?”: Qualitative exploration amongst older adults with diabetes with lived experience of surgical hospital admission

**Supplementary data**

Table of contents:

| **Appendix number and title** | **Page** |
| --- | --- |
| Appendix 1: Interview topic guide | 2 |
| Appendix 2: Preliminary codes, initial thematic framework and final themes and subthemes. | 3 |
| Appendix 3: How participant contributions informed the co-design process of the infographic | 4 |
| Appendix 4: Additional representative quotes | 8 |

# Appendix 1: Interview topic guide


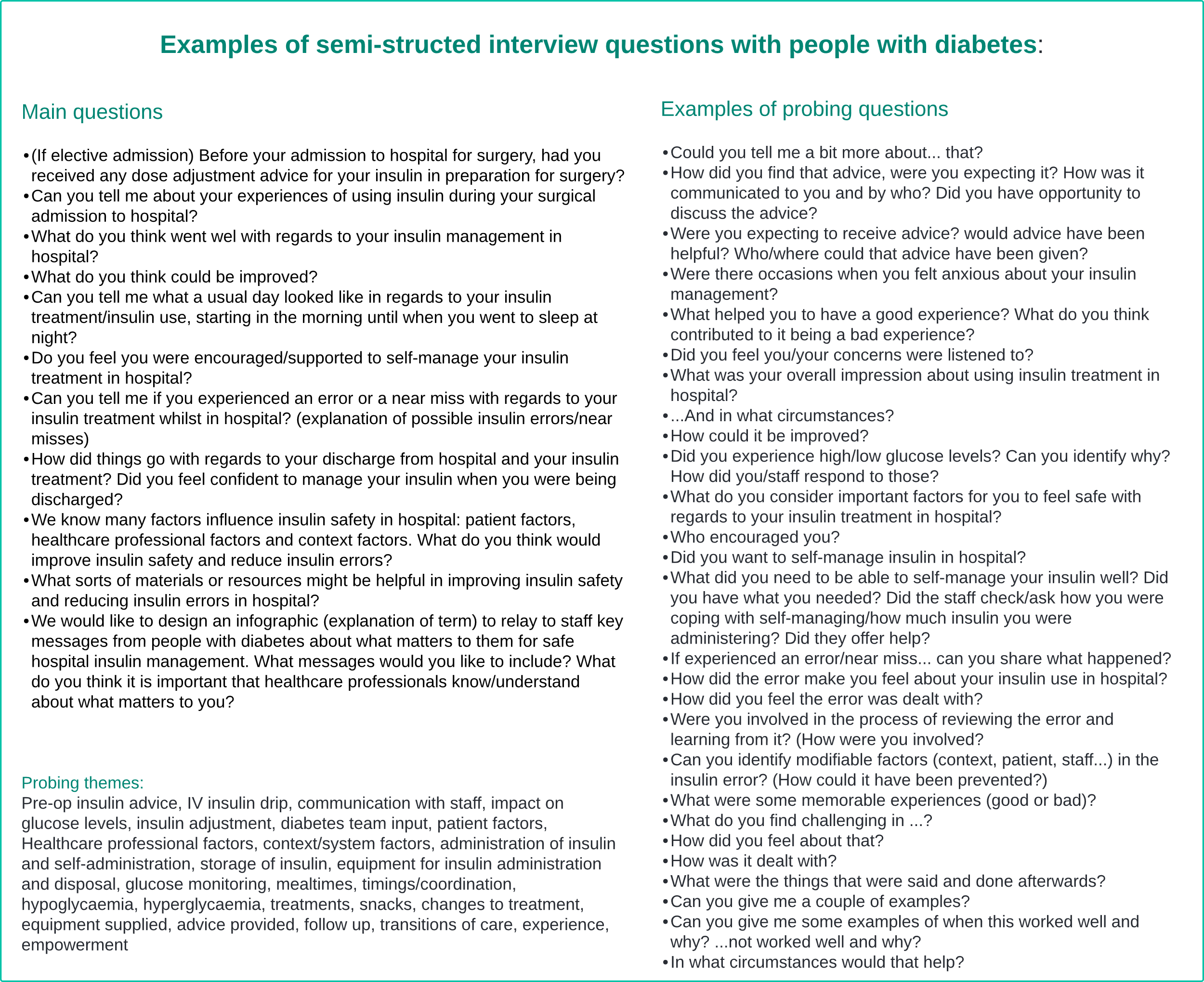


# Appendix 2: Preliminary codes, initial thematic framework and final themes and subthemes.

#
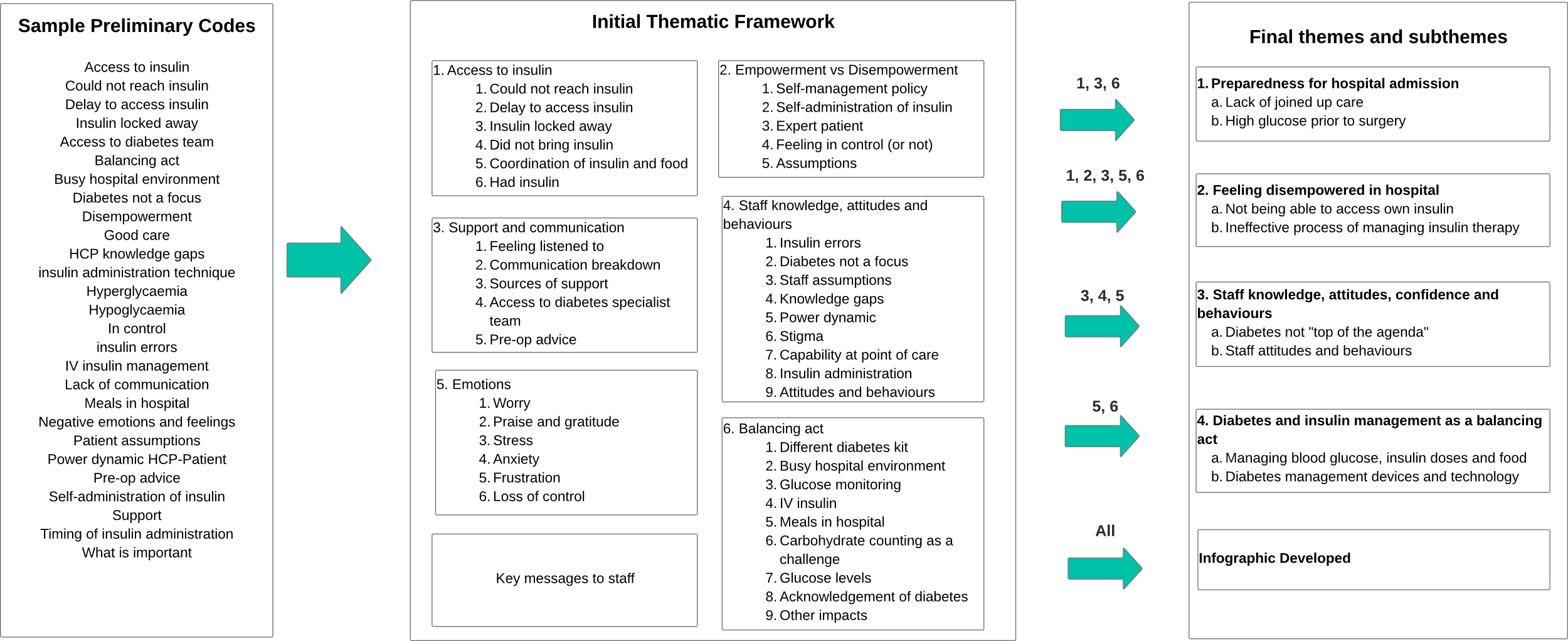


# Appendix 3: How participant contributions informed the co-design process of the infographic

# Early sketch


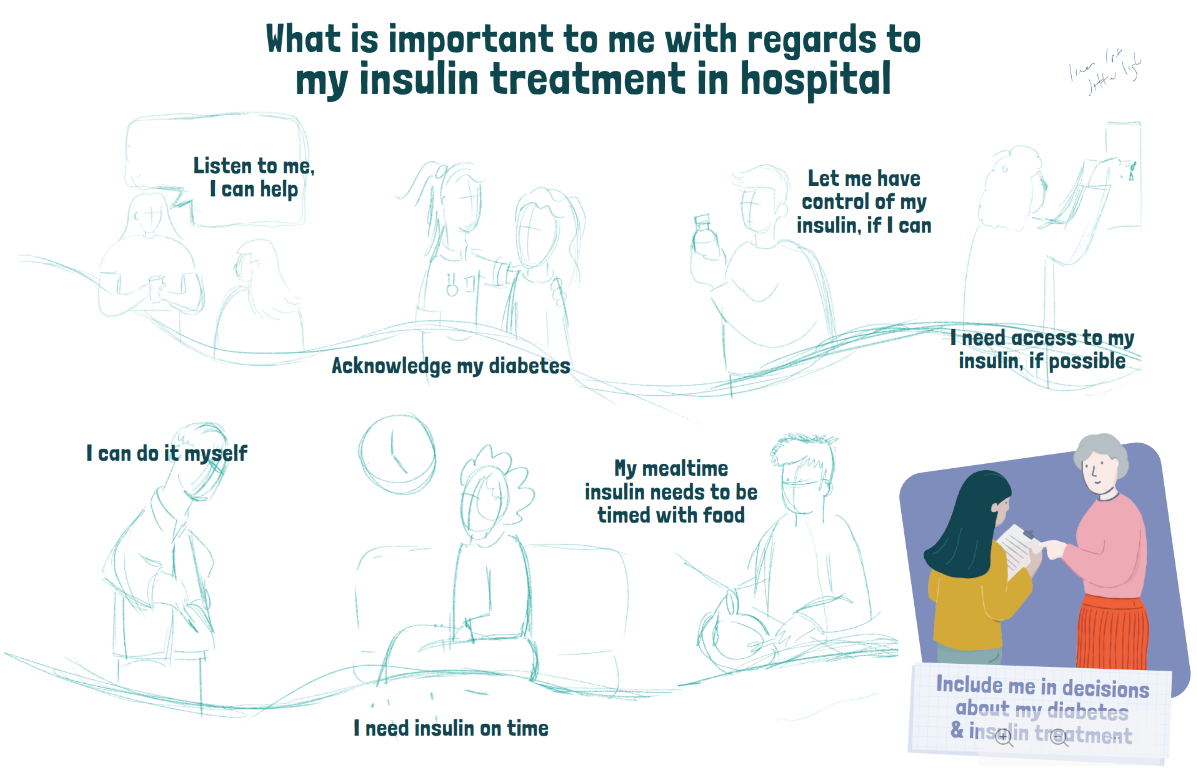


# Version sent in the post to all 10 interviewees


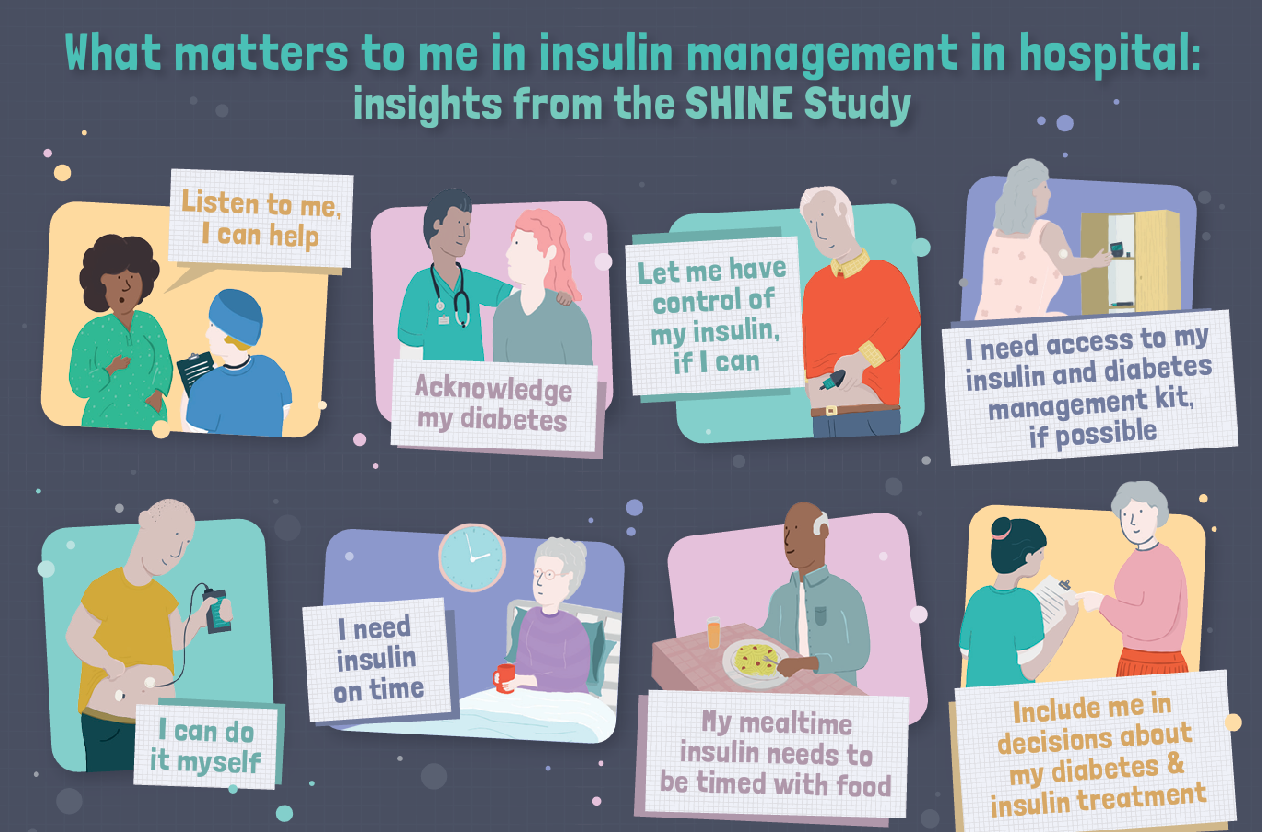


### 2.1 Structured feedback received from patients

Participants were sent by post a printout of the infographic and a structured feedback form and a pre-stamped return envelope. The form asked them to comment on the infographic and each of the eight illustrations and messages.

Sample of feedback form questions below:


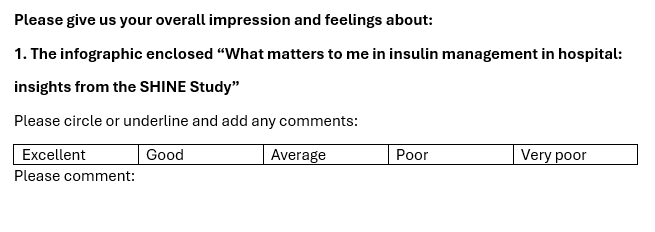


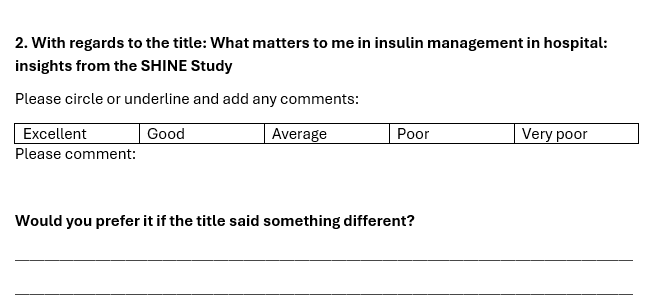


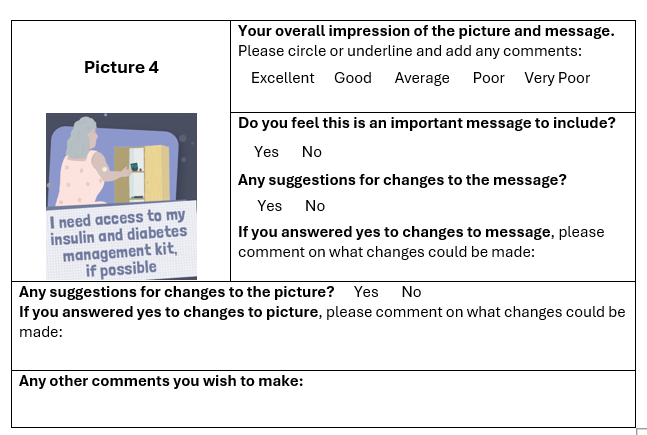


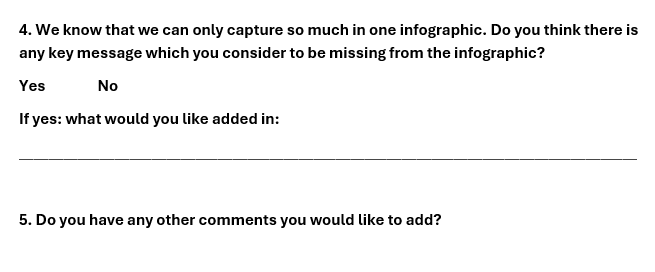


## 2.2 Examples of how feedback from patients informed refinement of the illustrations and messages within infographic:

### 2.2.1 Change in content of illustration and messaging (example below)


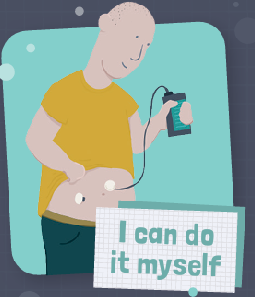

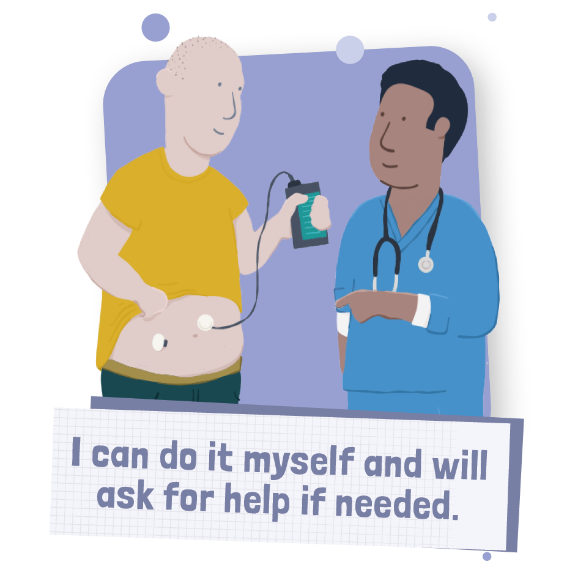


- Change to the message: I can do it myself, and will ask for help if needed.
- Change to the illustration: Addition of a healthcare professional in the illustration to frame decision for self-management of insulin in hospital as discussion and agreed plan between patient and healthcare professional, with possibility of support from staff if desire or ability to self-manage insulin fluctuated during the admission.

### 2.2.2 Addition of a new illustration (example below)


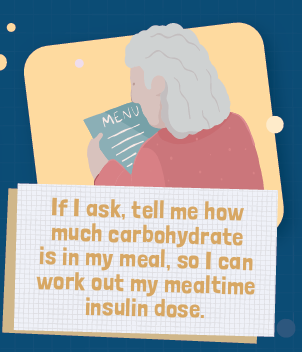


- The need to emphasise the importance of having information on carbohydrate content of meals for insulin management for those using mealtime insulin
- Merged 2 illustrations & messages on the need for insulin on time and mealtime insulin timed with meal into 1 illustration to provide space for the additional illustration regarding carbohydrate content

### 2.2.3 Change in the order of the illustrations (example below and see final version of infographic)

- The revised order with *"Let me have control"* at the start reinforcing patient autonomy from the outset.
- Grouping patient needs first and HCP actions later
- Change in order of HCP actions to *acknowledge, listen, include* sequence emphasising validation before action.

# Final version of infographic


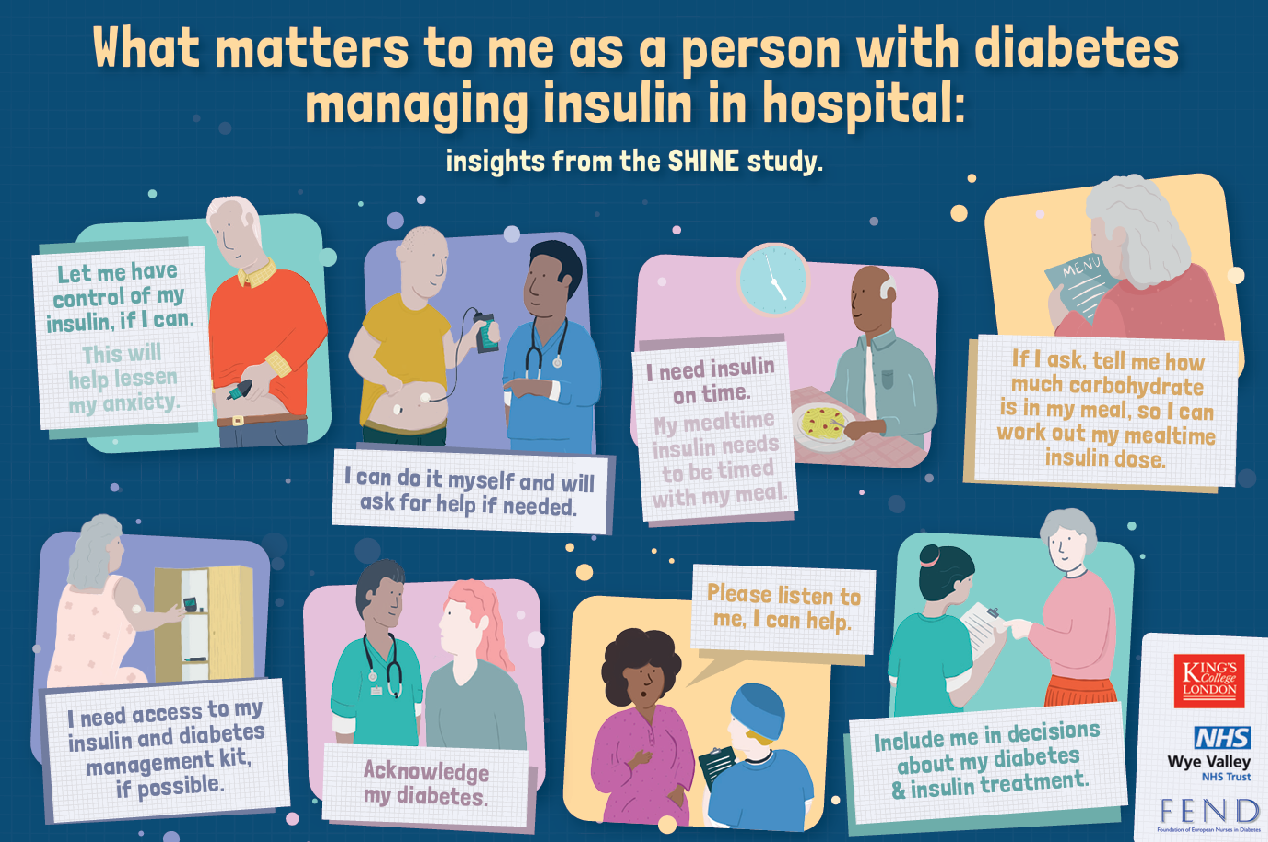


# Appendix 4: Additional representative quotes

| Theme | Representative quotes |
| --- | --- |
| Theme 1:  Preparedness for hospital admission | **Subtheme: Lack of joined up care**   - *“I was given a leaflet by the staff nurse when I came for pre-op assessment, (...) I don't recall there being a huge amount of advice on there.” (P4; 72 years old living with type 1 diabetes)* - *“I actually asked the anaesthetist [at appointment] whether I would need to be on a sliding scale (...) And… how can I put this? He just sort of emphasised that his job was to put me to sleep and basically, anything else would be another doctor." (P4; 72 years old living with type 1 diabetes)* - *“When I left the corridor downstairs to go up to the ward, (...) I did overhear what they were going to put on the form for my insulin. Which was 7.5, they’d said. I said, “That’s wrong. My reading here says about 15.2 or thereabouts.” And they said, “Oh, well, we’ve just tested you,” and I said, “No, you haven’t tested me at all, (...).so they put down the reading that I suggested on the form that goes up to the ward, which was 15.2. So, I got no hassle about it, but obviously a little bit of a mistake or misunderstanding on their part, (...) It must have been somebody else’s result that they had.” (P6; 85 years old living with type 2 diabetes)*   **Subtheme: High glucose prior to surgery**   - *"And when I checked my blood sugar in the room before going through for the operation it was quite high, which alarmed me (...) my stress level (laughs) went up quite a lot so my blood sugar level went up (...) Well, it never went back down again, it just stayed high.(...) In the late teens." (P3; 68 years old living with type 1 diabetes)* - *"I felt that if they’d have said to me, “Would you like an insulin drip?” I would definitely have said, “Yes, please” (P3; 68 years old living with type 1 diabetes)* - *“when I was admitted, my blood sugars were too high so they had to delay the op by an hour or two.(...) And it was all a little bit up in the air whether I went through with the surgery or not (…) “They gave me insulin [pre-surgery]. (...) a one-off [injection](...) And when I came round, they didn’t know whether to inject or not. (...) I don’t think they knew what to do with me at all actually.”* (P8; *74 years old living with type 2 diabetes*) |
| Theme 2: Feeling disempowered in hospital | **Subtheme: Not being able to access own insulin**   - *“So, I didn’t get to do my insulin. I asked about it and they said, “Oh, the nurse will be round with (...) your meds later,” and I was given something to eat and it just went on. And I kept saying, “I need my insulin,” and nobody was taking any notice. (...) in the evening, (...) they started to panic because my levels were at 27. (...) Well, I hadn’t had my insulin. I said, “You’ve locked it away and I can’t get to my medication”. (P5; 79 years old living with type 2 diabetes)* - *“The only time I sort of said, “Oh, I usually have this at six o’clock,” “Oh, well we do it when it’s the ward round or prescription time”. So, I said, “Okay”. (...)I just thought, well, (...) they’re trained nurses, they know what they’re doing. I had faith in them at the time.” (P8; 74 years old living with Type 2 diabetes)* - *"I think I was so ill, to be honest, I just… I think I just let it ride." (P10, 76 years old living with Type 2 diabetes)* - *“I was told to go in with all my medicines, which I would expect to do anyway because you need them all the time, but then to have them taken away from you… (...)it’s your mental ability to understand what’s going on, you know? ” (P3; 68 years old living with T1DM)*   **Subtheme: Ineffective process of managing insulin therapy**   - *"And I just felt completely, what's the word? Unempowered, disempowered? " (P4, 72 years old living with Type 1 diabetes)* - *“...I did fill a form in when I was in there to allow me to have access to my insulin at all times, but that didn’t seem to work.” (P2; 73 years old living with Type 1 diabetes)* - *“And in the end I said, “Why when I’ve been diabetic for 40 years can I not have control of my own insulin so that I can get my blood sugar down?” and they said, “It’s hospital policy you can’t do that” (P3, 68 years old living with Type 1 diabetes)* - *“I was sobbing my heart out when my granddaughter came to see me (...) and she was horrified. (...) She went and had a word with them and apparently there was a paper that if I had signed, nobody said anything, they never told me before I went in, that I could have had my insulin, I could have been in charge of it.” (P5, 79 years old living with Type 2 diabetes)* |
| Theme 3:  Staff knowledge, confidence, attitudes and behaviours | **Subtheme: Diabetes not top of the agenda**   - *“I mean, diabetes wasn't prominent in their minds, to be honest.(...) there obviously was no emphasis on whether or not they'd got a diabetic patient. And I don't mean that I would want to be singled out. It's just, feeling that they understand what I'm trying to do and hopefully let me get on with it."* (P4; 72 years old living with Type 1 diabetes) - *“Well, she [diabetes specialist nurse] was wonderful. And she sorted out the insulin, what to take, when and where, how much, so she altered it all. But that was on the day I was going.”* (P8; 74 years old living with Type 2 diabetes) - *“they hadn’t, (...) got a clue. I’m sorry, but they just didn’t. (...) because I haven’t got my insulin with me, that I can have some almost straight away, but they’ve got no access, you see, especially in A&E. I would have thought they would have given me something, even if it wasn’t Fiasp, you know, the old one, but no, no." (P1; 77 years old living with Type 1 diabetes)* - *“having to ask and then they checked again and said, “Yeah, that’s good,” but it [glucose] was quite high, so that was quite interesting." (P8, 74 years old living with Type 2 diabetes)* - *“(…) And I kept saying, “I need my insulin,” and nobody was taking any notice. (...) in the evening, (...) they started to panic because my levels were at 27[mmol/L]. (...) Well, I hadn’t had my insulin. I said, “You’ve locked it away and I can’t get to my medication”. (P5; 79 years old living with T2DM)*   **Subtheme: Staff attitudes and behaviours**   - *“He [the surgeon] noticed that I was on insulin. (...) And we discussed that and I said I gave the insulin myself. So, he said, “Well, right then, if that's the case,” he said, “I'm going to put you first on my [theatre] list”. (P7; 77 years old living with type 2 diabetes)* - *"He said he would give it [insulin] to me and I said no, I will give it. And he said, “Well, we perhaps might do”. I said, “No,” I said, “I'll give my own insulin”. I showed him what I was going to do and he was quite happy, quite interested in what I was doing and how I was doing it." (P7; 77 years old living with Type 2 diabetes)* - *“(...) the diabetes nurse specialists came up to the ward to see me. So, when I told them that nothing much seems to have changed, they went straight down to the nurses’ station and went through my notes and produced this signed form and explained to the nursing staff that this meant I should have access to my own insulin and control my own diabetes.” (P4, 72 years old living with type 1 diabetes)* - *“I felt very special actually, you know. And they used to come and always told me if they were going to do anything different and always came and had a chat every day." (P9, 91 years old living with Type 2 diabetes)* - *“the anaesthetist was brilliant (...) and he said, “Don’t worry, we’ll have it all under control and we’ll be keeping an eye on you”. And so he made me feel very, very reassured that I would be looked after properly, so I can’t praise him enough.” (P3; 68 years old living with Type 1 diabetes)* - *“Perhaps they just, it's taken at face value, you might be in your 70s, but the fact that you may have had it for the majority of your life, isn’t perhaps taken on board." (P4; 72 yearl old living with Type 1 diabetes)* - *"there was a nurse on night duty and I spoke to him about it and he said, “I'll go away and look for one of these forms”(...) which he duly did, and I was very grateful to him for that." (P4; 72 years old living with Type 1 diabetes)* - *"(...) I’m due to go back in (...) And I think at the back of my mind, I don’t want to be treated like I had been, make sense? (...) especially with my diabetic and insulin, you know, oh, whether it’s at the back of my mind the same thing’s going to happen again, that’s what I’m thinking.(...) I would have been more happy if I was more involved with my own insulin.” (P10, 76 years old living with type 2 diabetes)* |
| Theme 4  Diabetes and insulin management as a balancing act | **Subtheme: Managing blood glucose, insulin doses and food**   - *“let’s put it like this, I wasn’t sure what carbs to get. One of the diabetic nurses (...) said, would I like a printout of the menus with the carbs (...) and it was much easier for me to find, you know, the carbs for the meal...” (P1, 77 years old living with Type 1 diabetes)* - *“before they brought the food out, “how much insulin do you need to have?” And I said, “Well, I don’t know,” because I hadn’t seen what I was going to have yet, so I didn’t know how much to give myself.” (P2, 77 years old living with Type 1 diabetes)*   ***Subtheme: Diabetes management devices and technology***   - *"Well, I had the Libre, the Freestyle Libre, and none of the nurses appeared to have known about them (...) So when they came and did a test, we compared results, jokingly” (P6; 85 years old living with type 2 diabetes)* - *“But there again was a bit of a problem, I couldn’t understand it, (...) they had a completely different injection system to what I had. (...) which seemed to inject straight away. (...)They started to do it, yes, but I wasn’t happy, I didn’t think it had gone in or anything like that.(...) I had taken some extra needles in with me (...) on the third time I said, “No, I’d rather have my own needles,” and I injected myself and I injected myself in my stomach, as I normally do.” (P6; 85 years old living with type 2 diabetes)* - *"(...) you sit there and not panic, but I think well, if nobody can find needles for me to use, how am I going to give my insulin? (...) But I did get them some, and I can't remember exactly how. (Laughter)" (P4, 74 years old living with Type 1 diabetes)* |
